# An Extensive Unified Thermo-Electric Module Characterization Method

**DOI:** 10.3390/s16122114

**Published:** 2016-12-13

**Authors:** Filippo Attivissimo, Carlo Guarnieri Calò Carducci, Anna Maria Lucia Lanzolla, Maurizio Spadavecchia

**Affiliations:** Departement of Electrical and Information Engineering, Politecnico di Bari, Via Orabona 4, I-70125 Bari, Italy; filippo.attivissimo@poliba.it (F.A.); carlo.guarnieri@poliba.it (C.G.C.C.); anna.lanzolla@poliba.it (A.M.L.L.)

**Keywords:** thermoelectric modules, characterization, energy harvesting

## Abstract

Thermo-Electric Modules (TEMs) are being increasingly used in power generation as a valid alternative to batteries, providing autonomy to sensor nodes or entire Wireless Sensor Networks, especially for energy harvesting applications. Often, manufacturers provide some essential parameters under determined conditions, like for example, maximum temperature difference between the surfaces of the TEM or for maximum heat absorption, but in many cases, a TEM-based system is operated under the best conditions only for a fraction of the time, thus, when dynamic working conditions occur, the performance estimation of TEMs is crucial to determine their actual efficiency. The focus of this work is on using a novel procedure to estimate the parameters of both the electrical and thermal equivalent model and investigate their relationship with the operating temperature and the temperature gradient. The novelty of the method consists in the use of a simple test configuration to stimulate the modules and simultaneously acquire electrical and thermal data to obtain all parameters in a single test. Two different current profiles are proposed as possible stimuli, which use depends on the available test instrumentation, and relative performance are compared both quantitatively and qualitatively, in terms of standard deviation and estimation uncertainty. Obtained results, besides agreeing with both technical literature and a further estimation method based on module specifications, also provides the designer a detailed description of the module behavior, useful to simulate its performance in different scenarios.

## 1. Introduction

TEMs are increasingly being used for both power generation and for cooling/heating applications. In the first case the Seebeck effect is predominant to convert temperature gradient ΔT into electrical energy, whereas, in the second case, the dual effect—the Peltier effect—is exploited. TEMs’ several positive features make them reliable [1,2], and thus their use is appealing for both academic and industrial researchers.

In particular, Thermo-Electric Generators (TEGs) can generate clean energy in a small space and therefore are being used in an increased number of standalone applications ranging from aerospace [3,4], to industry [5,6,7]. Their possible integration with other energy harvesting technologies was also investigated; for example, for increasing the overall efficiency of photovoltaics [8] by exploiting the thermal gradient between the back of the panels and the ambient temperature, hence recovering the otherwise dissipated heat. The inherent restrained necessity of maintenance and the high long-term efficiency, make TEGs a valid alternative to batteries in providing an autonomous source of energy to sensor nodes [9,10,11] or entire Wireless Sensor Networks [12,13,14,15,16].

In addition, Thermo-Electric Coolers (TECs) have been considered, for a long time, for a number of applications such in that described in [17], where they are used to keep the chip temperature below an assigned threshold in electronic packaging systems, or in specialized air conditioning [18,19] and refrigeration applications [7,20,21,22].

Performances of a TEC are typically considered in correspondence to an optimal operating current that yields the maximum heat flux for a given operating condition, but in many cases, a TEC-based system is operated under the best conditions for only a fraction of the time, unless it is operated at a fixed heat load and fixed ambient conditions. Thus, the performance of a TEC at off-peak conditions are of great importance in the design phase and in determining the actual efficiency under dynamic working conditions.

However, manufacturers often only provide some essential parameters to characterize TEMs. Regarding the specifications of a TEC, some parameters are specified in correspondence of a couple of temperature values of the hot side (typically 27 °C and 50 °C). These usually are the largest temperature differential $\Delta T_{max}$ that can be obtained between the two sides along with the input direct current $I_{max}$ and the voltage $U_{max}$ that have produced it. Conversely, for TEG modules they usually provide the power $P_{e_{m}}$, the voltage $U_{m}$ and the maximum efficiency $\eta_{m}$ at matched load, i.e., when the load resistance $R_{m}$ equals the internal electrical resistance of the module. Clearly, these values do not correspond to actual ones but to maximum theoretical specifications, scarcely significant for practical use in many applications.

The need to quantitatively characterize TEMs’ behavior, implies the design of a robust testbed capable of addressing a twofold objective: to estimate the parameters of a TEM for design purposes and to define a robust method for comparing different TEM models. The development of a useful testbed for the characterization of TEMs is the main topic of this work. The focus is on using an appropriate procedure and suitable hardware to investigate the underlying relationship between both the operating temperature, the temperature difference and the parameters of the electrical and thermal equivalent model for commercial modules. The novelty consists in applying a proper current stimulus (for which two alternatives are discussed) to the TEM under test and simultaneously acquire both thermal and electrical data to automatically derive all parameters in a single test, without using either a discrete load [9,23,24] nor hot plates or auxiliary TEMs [2,11,17,24,25,26,27,28,29]. Unlike other approaches, the developed testbed permit, using a very simple configuration, to quickly obtain all relevant parameters, making possible an extensive TEM characterization over a wide range of temperature differences, ambient temperatures and electric loads.

This paper is structured as follows: after the introduction of the thermoelectric Figure of Merit Z, a typical method to estimate the thermal resistance which involves the measurement of four different temperatures between the surfaces of a reference material is described in Section 2.1. The state-of-the art review about electrical characterization is then described in Section 2.2 and subsequently the proposed method is briefly explained in Section 2.3. In Section 3 and Section 4 the electrical and thermal characterization are analytically approached, respectively, whereas in Section 5 the testbed is presented and detailed. Finally, results obtained with the two alternatives of the proposed approach are experimentally compared to each other in Section 6, confirming was expected from the analytical analysis. The obtained parameters are presented and validated with a third estimate based on the manufacturers’ specification [30]. Section 7 summarizes the paper’s content drawing the conclusions.

## 2. State of the Art and Proposed Method

The thermoelectric figure of merit Z is a parameter which summarizes the bulk material proprieties and allows the comparison among different TEMs. Alternatively, the same meaning is given to a dimensionless parameter $Z\bar{T}$ where $\bar{T}$ is the average absolute temperature between the two surfaces of the module. It is defined as:

(1) $$Z=\frac{\alpha_{S}^{2}\Theta_{IN}}{R_{IN}}$$

Expression (1) requires the knowledge of the relative Seebeck coefficient $\alpha_{s}$, the internal electrical resistance $R_{IN}$ and the thermal resistance $\Theta_{IN}$ of a TEM, all parameters that provide the most comprehensive description of the physical behavior of a TEM both as TEG and TEC. In fact, by recourse to them it is for instance possible to express the electrical power $P_{e}$ delivered to the load and the thermal power $P_{t}$ absorbed by the module in variable working conditions for a TEG module:

(2) $$P_{e}=V_{L}I=\frac{\alpha_{s}\cdot \Delta T\cdot R_{L}}{R_{IN}+R_{L}}\cdot \frac{\alpha_{s}\cdot \Delta T}{R_{IN}+R_{L}}\to P_{e_{m}}={{\left(\frac{\alpha_{s}\cdot \Delta T}{2\sqrt{R_{IN}}}\right)}^{2}|}_{R_{L}=R_{IN}}$$

(3) $$P_{t}=\Delta q=\frac{\Delta T}{\Theta_{IN}}$$

where $V_{L}$ is the voltage on the load. Furthermore, the efficiency η of a module, i.e., the amount of thermal power converted in electrical power, may be derived in all working conditions as:

(4) $$\eta =\frac{P_{e}}{P_{t}}=\frac{\alpha_{S}^{2}\cdot \Delta T^{2}\cdot R_{L}}{{\left(R_{IN}+R_{L}\right)}^{2}}\cdot \frac{\Theta_{IN}}{\Delta T}\to \eta_{m}={\frac{\alpha_{S}^{2}\cdot \Delta T\cdot \Theta_{IN}}{4R_{IN}}|}_{R_{L}=R_{IN}}$$

If Equations (2) and (4) are calculated at match load conditions (i.e., $R_{L}=R_{IN}$), it is possible to obtain the maximum value of the electrical power deliverable to the load $P_{e_{m}}$ (or equivalently the maximum power density if dividing it by the module area) and the maximum efficiency $\eta_{m}$ which are generally reported in datasheets.

Methods used nowadays to estimate such parameters suffer from various limitations, such as the necessity to realize different experiments to carry out all the requested measurements for Z calculation [31], or conversely, they directly provide it without letting one extract the single components [32]. Anyway, none of these is able to provide an extensive description of TEMs behavior in variable working conditions. In the following, a summary of these methods is reported.

### 2.1. Thermal Characterization

Among the above mentioned three parameters, the thermal resistance $\Theta_{IN}$ results, in principle, quite simple to be obtained but less immediate to be measured, since it requires different temperature probes and the realization of a suitable conditioning circuitry. It can be indirectly measured using a test configuration like the one described in Section 5, measuring the emitted and absorbed heat fluxes ($q_{em}$ and $q_{abs}$, respectively) and applying the thermal equivalent of Ohm’s law Θ=ΔT/q.

Once the fluxes are known, it is possible to write down the energy balance (5) between the Peltier effect, the heat conduction and Joule effect due to the injected current:

(5) $$\begin{array}{l} q_{em}=\frac{T_{3}-T_{4}}{\Theta_{ref}}=\alpha_{S}I_{st}T_{h}-\frac{T_{h}-T_{c}}{\Theta_{IN}}+\frac{I_{st}^{2}R_{IN}}{2}\\ q_{abs}=\frac{T_{2}-T_{1}}{\Theta_{ref}}=\alpha_{S}I_{st}T_{c}-\frac{T_{h}-T_{c}}{\Theta_{IN}}-\frac{I_{st}^{2}R_{IN}}{2} \end{array}$$

where $\Theta_{ref}$ is the thermal resistance of the reference medium. Its value can be previously fine-tuned sourcing the module with a known electrical power and solving the energy balancing equation $q_{em}-q_{abs}=VI$ with no thermal losses considered since they are negligible if the measurement setup is thermally isolated. Of course, it would be necessary to know the thermal dependence of $\Theta_{ref}$ for the adopted material with the mean temperature $\bar{T}$. However, if the investigated temperature range is not too wide, a material with an almost constant thermal conductivity in the given range may be adopted.

Therefore, in order to measure $\Theta_{IN}$, it is necessary to measure the current sourced to the module and both the emitted and the absorbed heat flux, which in turn require four temperatures to be measured or equivalently two voltage difference induced across a reference medium with a known thermal resistance $\Theta_{ref}$.

In Equation (5), $T_{h}$ and $T_{c}$ refer to those temperatures directly measured at the ends of the internal thermoelectric elements (pellets) and can be computed taking into account the temperature drops on the ceramic surfaces of a TEM:

(6) $$\begin{array}{l} T_{h}=T_{3}+q_{em}\Theta_{cer}\\ T_{c}=T_{2}+q_{abs}\Theta_{cer} \end{array}$$

where $\Theta_{cer}$ is the thermal resistance of the ceramic surfaces. However, in many cases, the use of alumina oxide (Al_2_O_3_) ceramic external plates in the module fabrication, makes the correction (6) negligible, since the joint combination of a high thermal conductivity (one order of magnitude higher than Bi_2_Te_3_) and a small thickness (one order of magnitude lower than Bi_2_Te_3_ pellets) lead to a thermal resistance $\Theta_{cer}$ value that is usually negligible as furtherly demonstrated in Equation (20).

Finally, solving Equation (5) with respect to $\Theta_{IN}$, it is possible to derive a measurement formula for the unknown thermal resistance:

(7) $$\Theta_{IN}=\frac{2\left(T_{h}-T_{c}\right)}{\alpha_{s}I_{st}\left(T_{h}+T_{c}\right)-\left(q_{em}+q_{abs}\right)}$$

where $\alpha_{s}$ is supposed to have been previously estimated, whereas $I_{st}$ is the steady-state current injected into the module under test.

This approach is very common and widely adopted when thermal characterization is required, using simple or more complex setups, depending on the application and uncertainty requirements. For example, in [24,25] the TEM is placed between a heat source given by a controlled hot-plate and a controlled cooling fan, whereas in [26] a shielded heating block and a cold-plate are used to assess the TEM efficiency, monitoring the heat flux, the delivered power and also the mechanical load to make a good thermal contact between each component. Another interesting setup is proposed in [27], where both the heat produced by an electrical resistance and the cooling capacity provided by a TEM, are controlled by a proportional-integral controller. Other propose the use of one or two opportunely driven auxiliary TEMs to set the temperature gradient on the TEM under test [2,28,33]. For example, in [28], $\Theta_{IN}$ is obtained measuring only one heat flux induced into an aluminum block with a known thermal conductivity, interposed between the module under test and an auxiliary TEM used to set the average temperature, hence requiring only three temperatures to be measured.

### 2.2. Electrical Characterization

At the end of fifties, Harman [34] developed the method, hence the name “*Harman Method*” (HM), used to obtain the constituent parameters of the thermoelectric figure of merit using both AC and DC measurements; the former to obtain the electrical resistivity whereas the latter to measure the Seebeck coefficient and the thermal conductivity. Generally, the HM is valid under the hypothesis of thermal equilibrium:

(8) $$T_{h}\approx T_{c}\approx T_{a}$$

In particular, when the temperature of the hot side $T_{h}$, the cold side $T_{c}$ and the ambient one $T_{a}$ do not deviate from the average temperature $\bar{T}$ by more than 0.3%, ensuring a good linear approximation of the radiative component in heat transmission, accurate to 1% [34]. This condition is typically achieved by injecting a current, large enough to be measured but sufficiently small (generally in the order of milliamperes) to meet the condition (8). In this condition it can be proved [34] that the figure of merit $Z\bar{T}$ can be written as $Z\bar{T}=V_{\alpha }/V_{r}$, where $V_{r}$ is the ohmic component of the voltage across the sample and $V_{\alpha }$ is the Seebeck voltage. In the testbed proposed by Harman, these two voltage components were alternatively measured immediately after switching a mechanical chopper on and off, responsible for the creation of a square-wave current. The main useful feature introduced by HM, was indeed the capability to estimate directly $Z\bar{T}$ in terms of a ratio of two voltages however, because of the technology limitations at the time, it suffered from a lack of precision and reproducibility [35].

**Figure 1:**
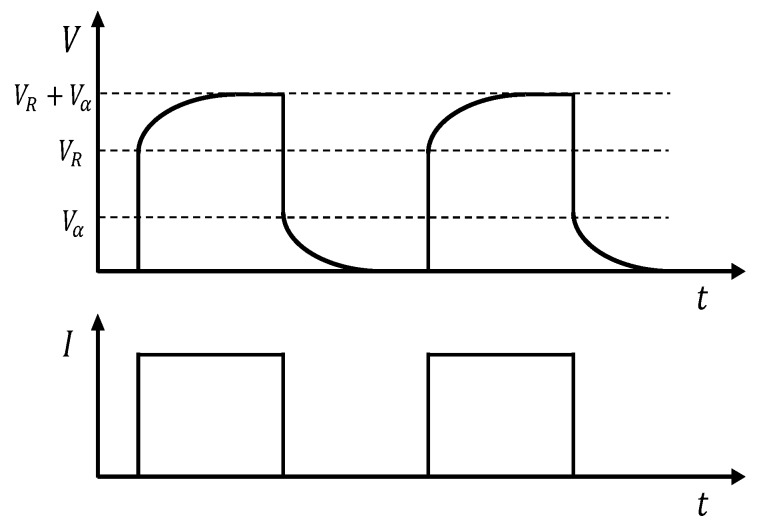
Typical TEM voltage profile using transient method: applied current *I* (**Bottom**) and resulting voltage *V* (**Top**) vs. time.

The method proposed by Harman was further developed by Buist in the so called “*Transient Method*” (TM) [35], in which the TEM is driven by a pulsed current like the one reported in Figure 1. At steady-state, the current is switched on, resulting in an immediate increase in the measured voltage because of the occurrence of the ohmic voltage component $V_{r}$. However, due to the slower characteristic response of heat phenomena compared to electrical ones, a temperature difference arises across the sample, generating a Seebeck voltage that slowly increases $V_{r}$ to $V_{r}+V_{\alpha }$.

The main difference between the HM and the TM is in the use of a modern instrumentation such as a DAQ board and a PC to reconstruct the entire voltage waveform over time (Figure 1). The use of the TM implies that, for each electrical working condition, a different current pulse have to be generated and acquired, but also that to have a good repeatability, a sufficient number of voltage waveform have to be processed.

The success of the TM is widely demonstrated by the large scientific literature concerning the TEM characterization that was inspired to it. Only to cite some: in [32], a test facility called “*Z-meter*” is proposed to measure the performance parameters of a TEM and some correction factors were introduced to take into account real environment conditions; in [28], the TM is used to estimate $\alpha_{s}$ and $R_{IN}$ in a commercial TEM. Another improvement with respect to the HM was proposed in [29], where both the temperature difference induced by applying a DC current and the heat flow created by a low frequency AC current, are used to obtain $\alpha_{s}$, $R_{IN}$ and $Z\bar{T}$, whereas $\Theta_{IN}$ is indirectly estimated using Equation (1). In [30] the HM is used to obtain the parameters of the TEM equivalent PSPICE model and to validate a method for extracting such model parameters from specifications. This latter feature is also used by the authors to derive a rough estimate of the TEM parameters and furtherly comparing them with the values obtained using the method proposed in this work.

### 2.3. Proposed Method

To overcome the limitations of the methods cited above, we propose a procedure which relies on two different modified versions of the TM for the electrical part while the typical approach described in Section 2.1 is used for the thermal characterization. The proposed method provides a detailed description of the three parameters that compose the figure of merit $Z\bar{T}$ with respect to variations of the operating conditions, i.e., the temperature of both sides of the module.

Briefly, the proposed method consists in placing the TEM module under test between two heat-flux sensors; when the desired operating conditions are met, a small signal or a rapid current variation is applied and the following quantities are measured: the emitted and the absorbed fluxes, the temperature at cold and hot side ($T_{h}$ and $T_{c}$), the voltage $V_{L}$ and the current I at its terminals. The developed testbed automatically sweeps along a wide range of drive currents using only a data-acquisition (DAQ) board and a 4-quadrant transconductance amplifier, but if the latter is not available, similar results may be obtained using the small signal method. Although all above parameters are measured at the same time, in the following section they are discussed separately to avoid confusion between electrical and thermal ones.

## 3. Electrical Measurement

A TEM can be electrically modelled as a voltage source $V_{th}=\alpha_{s}\Delta T$ in series with an internal resistance $R_{IN}$. Once the TEM reaches a steady-state condition, the voltage $V_{L}$ at its terminals is measured along with the current I flowing in it using a shunt resistor $R_{s}$ placed in series (described in Section 5). In this condition, the total voltage resulting at the power supply output terminals is:

(9) $$V\left(I,\Delta T\right)=V_{S}+V_{L}=R_{S}I+R_{IN}I+\alpha_{S}\Delta T$$

If a rapid current variation is applied, fast enough to not interfere with its thermal steady-state, then $V_{th}$ can be considered constant in such time window and the total voltage (9) is dependent only by the supplied current, hence on the electrical response of the module. Therefore, the resulting voltage at the terminals of the module under test is:

(10) $$V_{L}\left(I\right)=R_{IN}I+V_{th}↔\tau_{th}\gg \tau_{el}$$

To separate the two effects, this condition is obtained when the current variation occurs in a time window $\tau_{el}$ which is at least one order of magnitude smaller than the thermal time constant $\tau_{th}$.

### 3.1. Current Sweep (CS) Method

**Figure 2:**
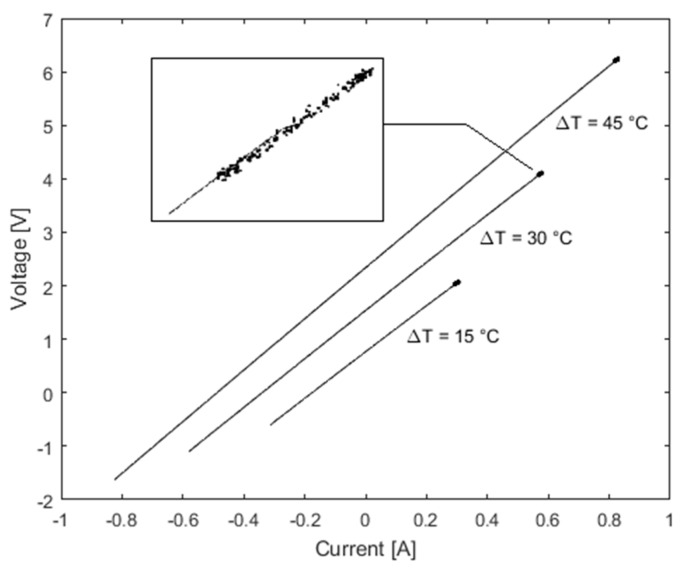
I−V plot for three different bias point of the CS (solid) vs. SS one (bold).

The adopted current variation consists in a CS from the value $I_{st}$ it has in the steady-state condition up to its opposite $-I_{st}$. Using Equation (10), this CS produces a line drawn in the I−V plane, as shown in Figure 2, where the slope is given by $R_{IN}$ and $V_{th}$ is its intercept with the ordinate axis, both referred to a given temperature $T_{c}$ and $T_{h}$.

### 3.2. Small Signal (SS) Method 

As described further in Section 4, the consistence of the CS method has been validated comparing it with a second current profile that produces similar results, but consists of a small sinusoidal signal $i_{ss}$ added to the steady-state current $I_{st}$, which can be considered to all effects as a perturbation around the bias point. From the theoretical point of view, such a small current does not require to satisfy condition (10) on the thermal time constant, since its amplitude is generally negligible with respect to $I_{st}$.

### 3.3. Analytical Comparison among CS and SS Method

Intuitively, the SS method would seem a better approach since it does not require any additional hypothesis, but the choice of a CS as current profile provides far more uniform results and a higher reliability in the estimation of both the involved parameters. This statement can be easily demonstrated analytically. Indeed, Equation (10) is a linear regression problem [36] where a given set of *n* pairs $\left(I_{i},V_{Li}\right)$ is used to find the best-fit line ${\hat{V}}_{Li}=V_{th}+R_{IN}I_{i}$, trying to minimize the sum of squared errors $SSE=\sum_{i=1}^{n}{\left(V_{Li}-{\hat{V}}_{Li}\right)}^{2}$. The regression slope $R_{IN}$, the intercept $V_{th}$ and respective standard deviations are computed using the following expressions [37]:

(11) $$R_{IN}=\frac{\mathrm{Cov}\left(I,V_{L}\right)}{\mathrm{Var}\left(I\right)}=\frac{\sum_{i=1}^{n}\left(I_{i}-\bar{I}\right)\left(V_{Li}-{\bar{V}}_{L}\right)}{\sum_{i=1}^{n}{\left(I_{i}-\bar{I}\right)}^{2}}$$

(12) $$V_{th}={\bar{V}}_{L}-R_{IN}\bar{I}$$

(13) $$\sigma_{R_{IN}}^{2}=\frac{s_{V_{L},I}^{2}}{SSI}=\frac{SSE}{\left(n-2\right)\left(n-1\right)s_{I}^{2}}$$

(14) $$\sigma_{V_{th}}^{2}=s_{V_{L},I}^{2}\left(\frac{1}{n}+\frac{{\bar{I}}^{2}}{SSI}\right)=\sigma_{R_{IN}}^{2}\left(\frac{\left(n-1\right)\cdot s_{I}^{2}}{n}+{\bar{I}}^{2}\right)$$

where $\bar{I}$ denotes the mean value of I, $SSI=\sum_{i=1}^{n}{\left(I_{i}-\bar{I}\right)}^{2}$ is the sum of I squared errors, $s_{I}^{2}$ is the sample, not population, variance of I and $s_{V_{L},I}^{2}$ is the error variance.

From Equation (14), it is immediate to see that the standard deviation $\sigma_{V_{th}}$ in the estimation of the intercept $V_{th}$ for a given $\sigma_{R_{IN}}$, increases linearly together with the samples standard deviation $s_{I}$ and with the mean value $\bar{I}$, which is always zero if a symmetric CS is used. Conversely, $\bar{I}=I_{st}$ when using a SS current profile and this produces higher errors when the value of the steady-state current is increased. Similarly, the variance $\sigma_{R_{IN}}^{2}$ in the estimation of the slope $R_{IN}$ is inversely dependent on the variance of I and increases as long as the samples tend to form a spot instead of spreading on the plane. This therefore means that using a symmetric CS, hence acquiring wider spread samples, leads to reduced errors in the estimation of fit parameters with respect to the SS method, but also that the CS method has a decreasing uncertainty in the slope estimation for increasing currents, whereas the SS method has a constant uncertainty.

After computing the standard deviations $\sigma_{V_{th}}$ and $\sigma_{R_{IN}}$, if the estimation errors are unbiased, normally and independently distributed, the 100(1−α)% confidence intervals on the slope and intercept can be expressed considering that the errors in the estimations of the same are both distributed as *t* random variables [38] with n−2 degrees of freedom:

(15) $$\begin{array}{l} u_{V_{th}}=t_{\alpha /2,n-2}\sigma_{V_{th}}\\ u_{R_{IN}}=t_{\alpha /2,n-2}\sigma_{R_{IN}} \end{array}$$

If the applied CS is not sufficiently fast to satisfy Equation (10), both because of an erroneous over-estimation of the module thermal time constant (Section 4) or because of intrinsic limits in the acquisition speed of the implemented test, a correction may be applied to the acquired signals. In fact, as shown further in Section 6, a quadratic term compares in the Kirchhoff’s voltage law equation, that induce a bending of the I−V line while the current varies. In this case, all the acquired values can still be used for parameters estimation, but the actual slope is the one at the beginning of the sweep, before the bending appears.

Hence, if Equation (10) is weakly satisfied, a linear regression with pure quadratic formula ${\hat{V}}_{Li}=a+bI_{i}+cI_{i}^{2}$ may be applied and the slope $R_{IN}$ and intercept $V_{th}$ may be computed as follows:

(16) $$\begin{array}{l} R_{IN}^{\prime }={\frac{dV_{Li}}{dI_{i}}|}_{I_{0}}\\ V_{th}^{\prime }=V_{L_{0}}-R_{IN}^{\prime }I_{0} \end{array}$$

where:

(17) $$\begin{array}{l} I_{0}=I_{st}\\ V_{L_{0}}=V_{Lst} \end{array}$$

## 4. Thermal Characterization

**Figure 3:**
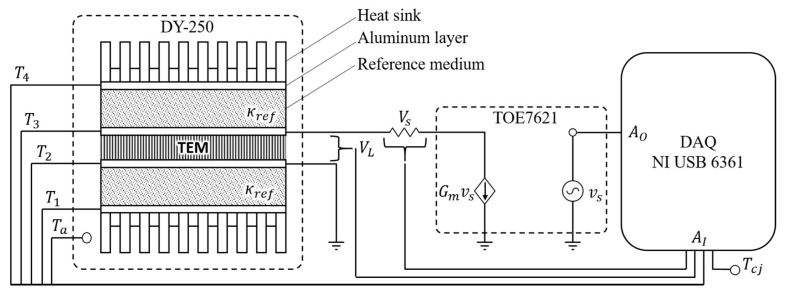
Automatic measurement setup.

Thermal measurements have been carried out using two heat flux sensors while varying the temperature of the two sides sourcing the module with an adequate current (Figure 3). Each one was implemented by the authors, as described in a previous work [38], stacking two metal layers (2 mm thick) for temperature sensing with an interposed medium with known thermal conductivity $k_{ref}$, both with the same geometric area of the module under test. In each metal layer, a small hole was made to accommodate the thermocouple sensors, whereas the contact resistance between layers was minimized using a thin layer of high thermal conductivity silver-based thermal paste. In order to satisfy Equation (10), the thermal transfer function of the module have to be preliminarily estimated; in this work it was used an Autoregressive Exogenous method included in the MATLAB^®^ System Identification Toolbox [39].

After applying a current step I(t), four temperatures $\left(T_{1},T_{2},T_{3},T_{4}\right)$ are measured and composed to create two differential temperature signals:

(18) $$\begin{array}{l} \Delta T_{41}\left(t\right)=T_{4}-T_{1}\\ \Delta T_{32}\left(t\right)=T_{3}-T_{2} \end{array}$$

where $\Delta T_{32}$ should be most representative of the response of the TEM itself, whereas $\Delta T_{41}$ should also take into account the response of entire system of which it is part. Equations (15) are then used to estimate two transfer function, expressed as the Laplace transform of a first order system:

(19) $$H_{\Delta T}\left(s\right)=\frac{ℒ\left[\Delta T\left(t\right)\right]}{ℒ\left[I\left(t\right)\right]}=\frac{A}{s+\frac{1}{\tau_{th}}}$$

where $A\tau_{th}$ is the DC gain.

The output differential temperature time constant at external sides of the heat-flux sensors is always greater than the one at direct contact with the TEM because of the heat propagating time in a given medium (i.e., $\tau_{th_{41}}\gg \tau_{th_{32}}$). Thus, $\tau_{th_{32}}$ is taken into consideration to establish the maximum temporal duration of the electrical perturbation $\tau_{el}$.

During the CS, all the four temperatures are acquired and elaborated according to the procedure described in Section 2.2 to compute the internal thermal resistance of the module $\Theta_{IN}$.

Since, even in the worst examined case (i.e., in correspondence of the highest heat fluxes), the temperature drops on the ceramic plates of the module (20) are negligible, it results that that $T_{c}\approx T_{2}$ and $T_{h}\approx T_{3}$ as previously stated in Equation (6):

(20) $$\begin{array}{l} \left|\frac{T_{3}-T_{h}}{T_{h}}\right|<0.3\%\\ \left|\frac{T_{2}-T_{c}}{T_{c}}\right|<0.007\% \end{array}$$

Making also explicit in Equation (4) the dependence of the remaining parameters on the acquired temperatures, we can rewrite the thermal resistance equation as a function of the measured variables as:

(21) $$\Theta_{IN}=\frac{2{\left(T_{2}-T_{3}\right)}^{2}}{V_{th}I_{st}\cdot \left(T_{2}+T_{3}\right)+k_{ref}\left(T_{1}-T_{2}-T_{3}+T_{4}\right)\left(T_{3}-T_{2}\right)}$$

where $k_{ref}=1/\Theta_{ref}$ is the thermal conductivity of the reference medium, obtained as described previously in Section 2.1 as calibration factor, minimizing the squared error associated to the flux/power balancing equation. Therefore, it is necessary to adopt a material that exhibits a low dependence of its thermal conductivity with the temperature in the investigated range.

At this point, if the following simplifying assumptions are made:

- $k_{ref}$ has no associated uncertainty since it is a calibration factor
- the four temperatures have the same uncertainty $u_{T}$ because they are measured with the same kind of temperature sensor (type-J thermocouple), they are acquired using the same DAQ board and referred to the same cold-junction sensor
- all variables are uncorrelated, except $I_{st}$ and $V_{th}$ that are obviously correlated

The uncertainty $u_{\Theta_{IN}}$ on the estimation of the internal thermal resistance can be derived applying standard uncertainty propagation [40] to Equation (21), hence, the thermal resistance uncertainty may be expressed as:

(22) $$u_{\Theta_{IN}}=\frac{\Theta_{IN}^{2}}{2\Delta T_{32}^{2}}\left|\bar{T}\left(V_{th}u_{I_{st}}+I_{st}u_{V_{th}}\right)+2V_{th}I_{st}u_{T}\right|$$

from which is mainly observable a clear quadratic inverted trend with respect to $\Delta T_{32}$.

## 5. Automatic Test 

The implemented automatic test procedure performs the above described measurement over a matrix of testing points obtained from the combination of two vectors containing respectively the desired temperatures for the cold side $T_{c}$ and the desired working ΔT. The whole test is conducted inside a Discovery Es 250 (DY-250) climate chamber (Figure 3) by Angelantoni Group S.p.A. (Massa Martana (PG), Italy), which forces the cold side of the module to follow the inner ambient temperature.

All measured variables, outlined below, are voltage signals acquired at $f_{s}=160\;\mathrm{Hz}$ by means of a 16-bit DAQ board X Series USB 6361 by National Instruments (Austin, TX, USA); in particular:

- $V_{L}$ is the voltage at the TEM terminals
- $V_{s}$ is the voltage across the shunt resistor $R_{S}$
- $V_{a}$ is the climate chamber ambient temperature measured by means of a LM35 temperature sensor (Texas Instruments, Dallas, TX, USA)
- $V_{1},V_{2},V_{3},V_{4}$ are the voltages generated by the four different J-type thermocouples each one inserted into one surface of the heat-flux sensors
- $V_{cj}$ is the cold junction compensation temperature acquired by means of a further LM35 sensor placed close to the DAQ board

The same DAQ board is also responsible for generating the driving voltage for a 4-quadrant TOE7621 transconductance amplifier (TOELLNER Electronic Instrumente GmbH, Herdecke, Germany) that supplies the TEM under test.

**Figure 4:**
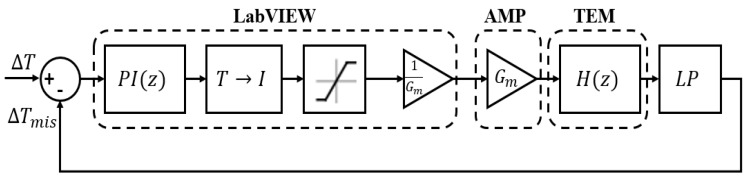
TEM control loop.

The entire acquisition/generation process is handled by a LabVIEW Virtual Instrument (VI) that runs a proportional-integrative (PI) controller (Figure 4) in a closed-loop feedback to drive the module to the desired steady state working conditions. Once known the current-to-thermal transfer function $H_{\Delta T}\left(s\right)$ estimated in Equation (19), the tuning parameters for the PI controller have been computed using both the closed-loop Ziegler-Nichols method and the MATLAB^®^ Simulink Auto-tune tool. Although both methods produce good results, the latter has been chosen because of its ability to produce a desired response in a smaller time with less overshoot.

**Figure 5:**
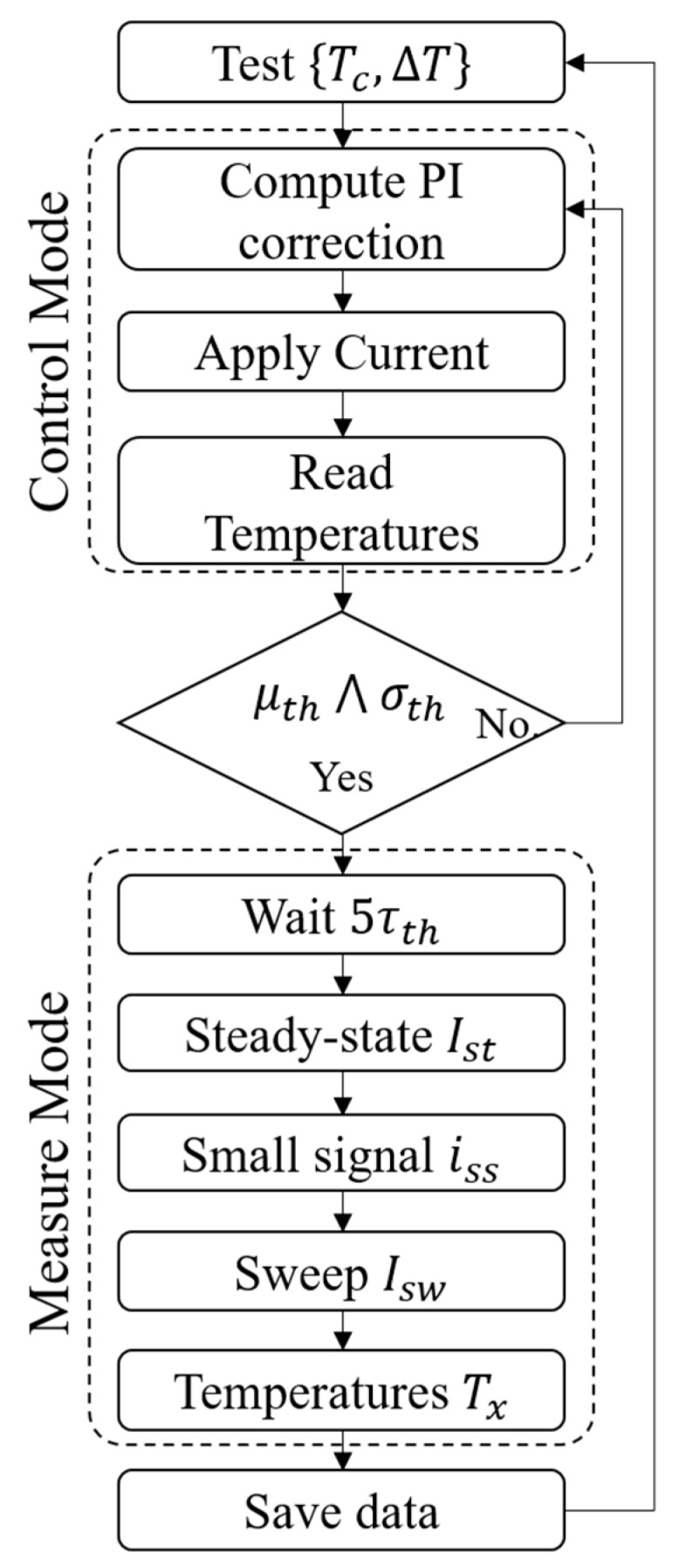
Test procedure flowchart.

The steady-state conditions for a given combination of $T_{c}$ and ΔT are met when the following two conditions are satisfied over 100 s or 16,000 samples:

(23) $$\begin{array}{l} \left|\mu_{\Delta T}-\Delta T\right|<\mu_{th}=0.15\;{}^{\circ}C\\ \sigma_{\Delta T}<\sigma_{th}=0.05\;{}^{\circ}C \end{array}$$

where $\mu_{\Delta T}$ and $\sigma_{\Delta T}$ are the mean and standard deviation of the measured temperature gradient, whereas $\mu_{th}$ and $\sigma_{th}$ are the respective threshold values. In this case, the VI locks the driving current to the last current value, waits for a time equal to five thermal time constant ($\tau_{w}=5\tau_{th_{41}}$) to allow all transients to run out and then switches from Control Mode to Measure Mode (Figure 5).

**Figure 6:**
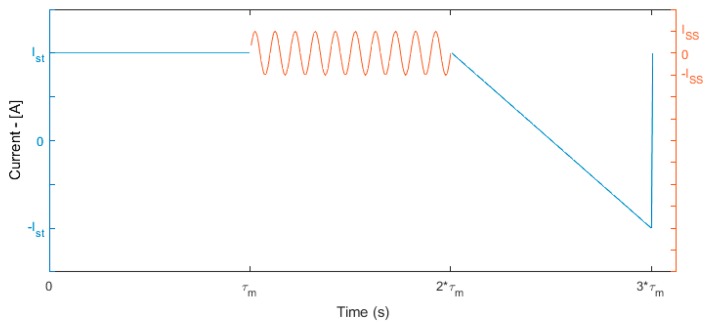
Measurement procedure signal time pattern.

The measurement procedure consists of three sections run in sequence (Figure 6), in which three different current profile are generated, each one with a duration $\tau_{m}$ that is set to a value sufficiently small with respect to the module thermal time constant ($\tau_{m}\leq \tau_{el}\ll \tau_{th}$):

(1) *steady-state:* the last driving current value is applied and the acquired values are used to compute statistics over $I_{st}$.
(2) *SS:* a small current 10 Hz sinusoidal stimulus is added to $I_{st}$, with an amplitude equal to the ratio of standard deviation threshold to the static gain of Equation (19):
  (24) $$I_{SS}=\frac{\sqrt{2}\sigma_{th}}{H_{\Delta T}\left(0\right)}$$
(3) *CS:* the driving current $I_{st}$ is swept to its symmetric value $-I_{st}$ with a ramp-like signal that is finally switched back to the initial value.

At the end of each test, all raw data (acquired voltage signals) are stored for further post-processing using MATLAB:

- $V_{L}$ is left as it is.
- $V_{s}$ is divided by $R_{s}$ to obtain I.
- $T_{a}$ and $T_{cj}$ are obtained from respective voltages using the LM35 nominal sensitivity *S* = 10 mV/(°C).
- $T_{1},T_{2},T_{3},T_{4}$ are computed using the coefficients provided by National Institute of Standards and Technology (NIST) and applying a software cold-junction compensation.
- $q_{em}$ and $q_{abs}$ are computed as described in Section 2.1.
- $T_{c}$ and $T_{h}$ are derived by $T_{2}$ and $T_{3}$ computing the temperature drop on the ceramic layers induced by the computed heat fluxes.

Once all the raw signals have been properly scaled, the three parameters composing the thermoelectric figure of merit $Z\bar{T}$ can be calculated for each working condition $\left\{T_{c},\Delta T\right\}$:

- $V_{th}$ and $R_{IN}$ are derived using Equation (10), applying a linear regression to acquired values $\left\{I,V_{L}\right\}$.
- $\alpha_{s}$ is then obtained as ratio of $V_{th}$ to ΔT.
- $\Theta_{IN}$ is computed using Equation (7).

## 6. Experimental Results

The above described procedure has been used to characterize the performance of a commercial low-cost module TES1-12730 from Thermonamic Electronics Corporation (Nanchang, China) [41]. For sake of clarity, the experimental results are split in sub-sections: firstly, the results of the module identification procedure will be presented, then the consistence of the SS method is shown and finally the CS method is validated and characterization results will be shown.

### 6.1. Module Identification

**Figure 7:**
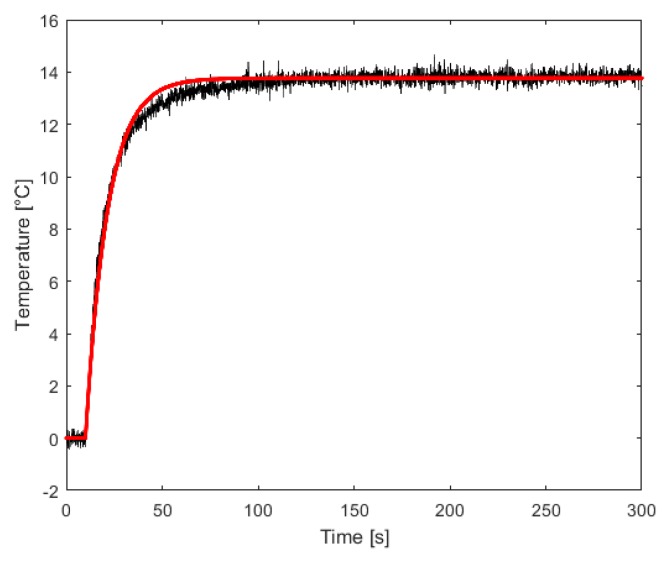
Measured step response $\Delta T_{32}$ (black) vs. simulated (red) of the identified model $H_{32}$.

The TEM has been identified applying a 0.1 V voltage step to the amplifier, which results in a 320 mA current step because of the transconductance gain $G_{m}=3.2$ A/V. The measured differential output signals $\Delta T_{32}$ and $\Delta T_{41}$ are thus used to obtain the two respective transfer functions, which continuous-time models are resumed in Table 1; $H_{32}$ (Figure 7) is then used to tune the feedback loop in Figure 4.

Once the PI controller was tuned, the module was tested over 210 different working conditions $\left(T_{c},\Delta T\right)$ with a 14 × 15 sampling matrix, where $T_{c}\in \left[10\;49\right]$ °C and ΔT∈[3 45] °C (with 3 °C steps), for a whole duration of approximately 52 h.

Before starting the test, the adopted value of the time test window $\tau_{m}=1\;s$ has been considered sufficiently small compared with the thermal time constant $\tau_{th_{32}}=11.5\;s$ of $H_{32}$. This indeed have produced $N_{s}=\tau_{m}f_{s}=160$ samples for each measurement section.

### 6.2. SS Method Analysis

**Figure 8:**
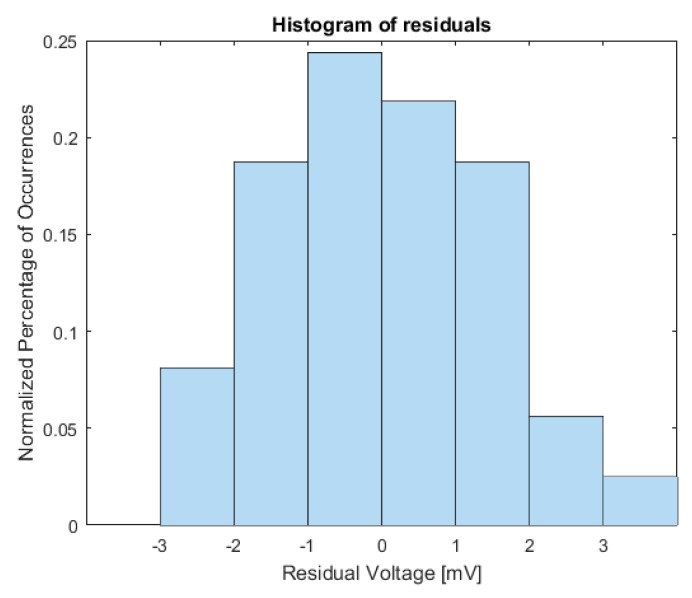
Residuals of linear regression model.

After elaborating the acquired data for the SS current stimulus, statistics have shown the consistence of this measurement method. The linear regression model associated with Equation (10), for each of the 210 tests, was estimated with a mean value of the determination coefficient ${\bar{R}}^{2}=0.99550$ and a standard deviation $\sigma_{{\bar{R}}^{2}}=0.00023$. If we consider the test with the worst regression fit ($R^{2}=0.9919$) relative to $T_{C}=49$ °C and ΔT=45 °C, the plot of residuals (Figure 8) shows that they are, in any case, normally distributed with zero mean.

**Figure 9:**
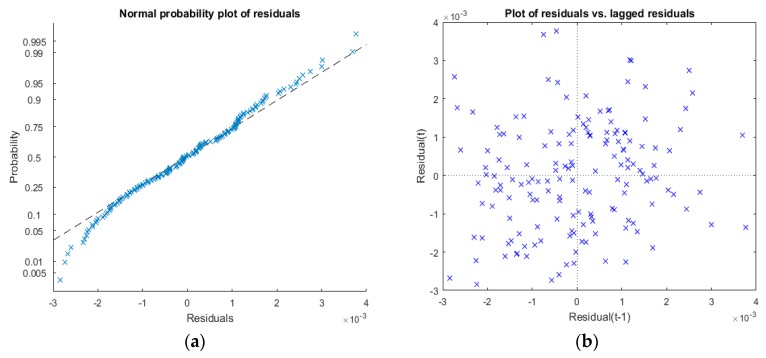
(**a**) Normal probability plot of the residuals of the fitted model; (**b**) Plot of residual vs. lagged residuals for serial correlation analysis.

Furthermore, the probability plot in Figure 9a, shows how the distribution of the estimation residuals compared with a normal distribution with matched variance, confirms a reasonable fit to normally distributed residuals with almost no evident outliers. The residual lag plot (Figure 9b) also shows the absence of any remaining serial correlation among residuals, since they are all distributed uniformly among the four quadrants.

**Figure 10:**
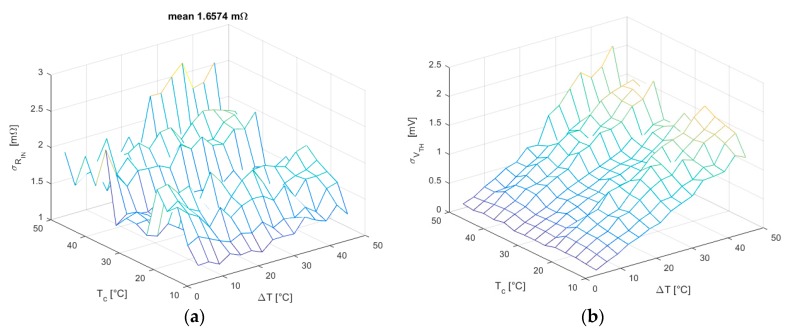
(**a**) $R_{IN}$ standard deviation in all working conditions for SS method; (**b**) $V_{th}$ standard deviation in all working conditions for SS method.

Hence, the SS method seems to satisfy all demands for the application of the linear regression and the confidence intervals relative to the estimated parameters may be expressed as in Equation (15). In Figure 10, the $R_{IN}$ and $V_{th}$ standard deviations obtained using Equations (13) and (14) are reported for all working conditions, showing that the $\sigma_{R_{IN}}$ is almost constant through all tests. In fact, as explained in Section 3.3, it mainly depends on the variance*s* $s_{I}^{2}$ of I data, which is set to a constant value in Equation (24). Similarly, the linear trend in the intercept standard deviation is due to its dependence on the mean value of I data, which increases for increasing ΔT.

### 6.3. CS Method Analysis

Applying to CS data the same procedure just described, the obtained main determination coefficient is ${\bar{R}}^{2}=0.999965$ with a standard deviation $\sigma_{{\bar{R}}^{2}}=2.93\times 10^{-6}$.

**Figure 11:**
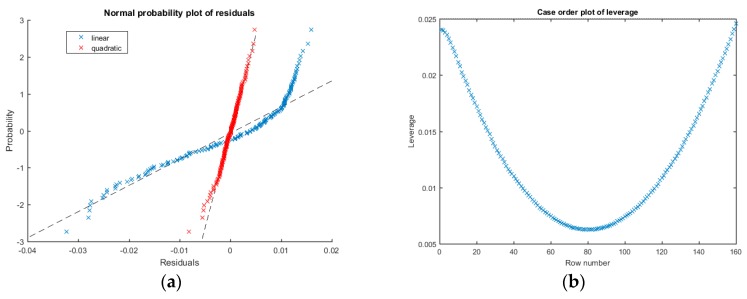
(**a**) Normal probability plot of the residuals of the fitted model: linear (blue) and quadratic (red); (**b**) Leverage plot of data and model

Despite of the high value of ${\bar{R}}^{2}$, the residuals of the linear regression for the same working point analyzed before (i.e., $T_{c}=49$ °C and ΔT=45 °C), show a non-zero mean and a clear non-normal distribution as evinced from Figure 11a, which means that a linear fit is not able to fully explain the underlying data relation. This is probably due to the fact that the adopted measure time window $\tau_{m}$, even if one order of magnitude smaller then thermal time constant, is too large to satisfy the hypothesis in Equation (10). Nevertheless, a visual inspection of residuals plot (Figure 11b) shows a clear quadratic relation, which suggests to fit the data using a non-linear formula.

Hence, applying the least squares method with a quadratic formula as described at the end of Section 3.3, better fit results are obtained (Figure 11a) with a resulting mean determination coefficient ${\bar{R}}^{2}=0.999996$ and a standard deviation $\sigma_{{\bar{R}}^{2}}=2.22\times 10^{-6}$. Also, the resulting residuals mean error is zero and these are normally distributed, confirming a successful fit.

**Figure 12:**
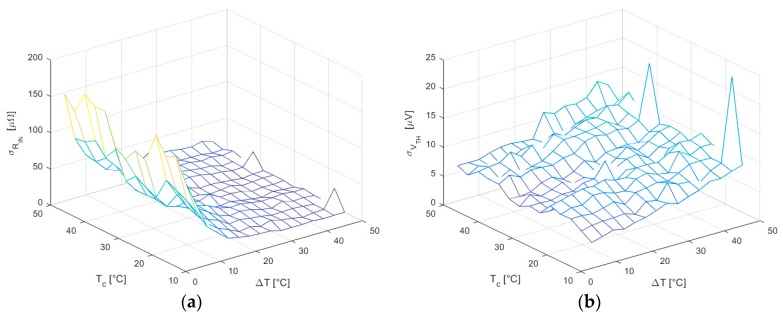
(**a**) $R_{IN}$ standard deviation in all working conditions for CS method; (**b**) $V_{th}$ standard deviation in all working conditions for the CS method.

As mentioned in Section 3.3, the CS method produces far more uniform results with much lower uncertainties, as showed in Figure 12.

Since when using this method, the variance of I is not constant and increases for higher ΔT, a clear downward trend occurs in $\sigma_{R_{IN}}$; whereas $\sigma_{V_{th}}$, which depends both on $\bar{I}$ and $s_{I}^{2}$, shows a growing trend because of the increment of the latter, while the former tends to zero due to the sweep symmetry.

**Figure 13:**
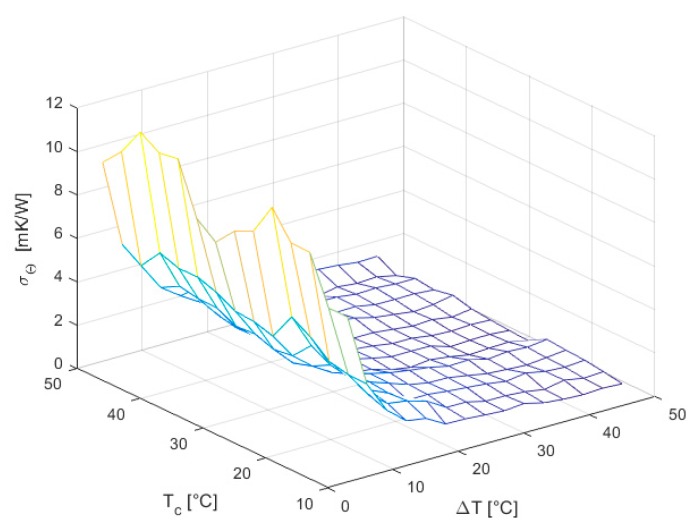
Standard deviation of thermal resistance $\Theta_{IN}$.

Conversely, the standard deviation of the thermal resistance $\sigma_{\Theta_{IN}}$ (Figure 13) follows a trend similarly to $\sigma_{R_{IN}}$ as expected from Equation (22).

### 6.4. Experimental Comparison among CS and SS Method

In order to test the compatibility among the CS and SS method, for all working conditions, a point by point relative error Equation (25) is computed and mean values as well as standard deviations are reported in Table 2:

(25) $$\begin{array}{l} e_{r}=\frac{P_{CS}-P_{SS}}{P_{SS}}\\ {\bar{e}}_{r}=\frac{1}{n_{i}n_{j}}\sum_{i\in T_{c}}\sum_{j\in \Delta T}\frac{P_{CSij}-P_{SSij}}{P_{SSij}} \end{array}$$

where $P_{xx}$ represent the generic estimated TEM parameter (i.e., $\alpha_{S}$, $R_{IN}$, $\Theta_{IN}$ or $Z\bar{T}$).

Computed mean relative errors show a good match between the two methods, with halved error values for CS method using a quadratic formula with respect to SS method and a linear regression.

**Figure 14:**
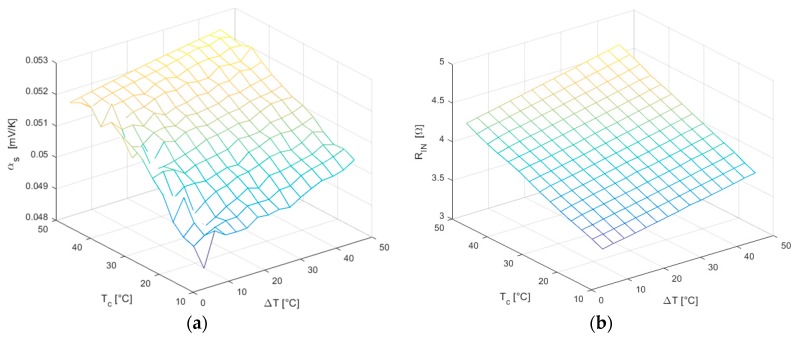
(**a**) Seebeck coefficient $\alpha_{S}$; (**b**) Internal electrical resistance $R_{IN}$.

The result of the characterization process of the module TES1-12730, using the proposed CS method, produced interesting results. Both the Seebeck coefficient $\alpha_{S}$ (Figure 14a) and the internal electrical resistance $R_{IN}$ (Figure 14b) increase their value almost linearly as long as $T_{C}\propto \bar{T}$ and ΔT increase. Despite the Seebeck voltage appears smooth and uniform, the trend of the Seebeck coefficient shows to be more irregular because it is derived as ratio of the former to the temperature difference ΔT, which is less accurate for small values.

**Figure 15:**
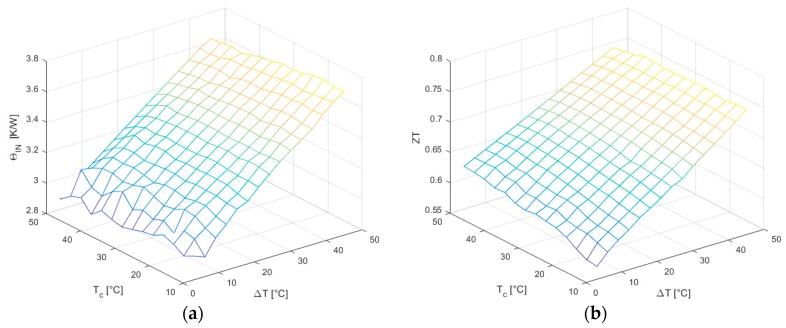
(**a**) Internal thermal resistance $\Theta_{IN}$; (**b**) Figure of merit $Z\bar{T}$.

Regarding the internal thermal resistance $\Theta_{IN}$ (Figure 15a), it shows a strong dependence with increasing ΔT, whereas it seems to be only slightly affected by variations of $T_{C}$, which decrease induces an observed weak resistance increment. The roughness of the surface for small ΔT is due also in this case to its analytical dependence on measured temperatures.

The figure of merit $Z\bar{T}$ (Figure 15b) exhibits a strong linear dependence on ΔT and a weaker one on $T_{C}$, and since it is a combination of the previous mentioned parameters, it also shows a bending that is taken into account using second order terms in the interpolating function.

## 7. Conclusions

The novelty of the proposed technique consists in using a simple testbed to stimulate the module and to simultaneously acquire electrical and thermal data so to obtain the three parameters that compose the figure of merit *Z*. An extensive characterization of TEMs in many variable working conditions is hence performed within a single test.

The developed testbed automatically sweeps along a wide range of drive currents using only a DAQ board and a 4-quadrant transconductance amplifier, but if the latter instrument is not available, the authors have shown that similar results may also be obtained using a different approach.

To validate the method, we firstly compared the results obtained using two different current profiles: a small signal one that require no previous assumption and a sweep signal that conversely requires a preliminary hypothesis to be satisfied, but that produce far more accurate results; furtherly we compared them with those provided in the datasheet by the manufacturer. The performance of both approaches was theoretically analyzed and experimentally verified, demonstrating a good match between the two methods through a comparison among data generated by CS and SS profiles.

If using the current sweep approach, the authors have shown that obtained results may be furtherly improved without redoing all the tests, but simply applying a numerical correction to acquired data.

The experimental results (i.e., $\alpha_{S}$, $R_{IN}$, $\Theta_{IN}$ and $Z\bar{T}$) for variable ΔT and $T_{C}$ working conditions have been reported in exhaustive tri-dimensional graphs, which values (reported in Table 3) are in agreement with those extrapolated from the datasheet using a different method.

Finally, simple analytical relations were derived from interpolation of obtained data and reported in Table 4 to provide a simple tool for eventual comparison with other data and methods and to simulate the TEM performance in different scenarios.

The reported modeling at different operating conditions, may play an important role in estimate the performance of TEMs for energy generation, affecting for instance the harvestable energy as long as the internal electrical resistance moves away from designed matched value.

## Figures and Tables

**Table 1:** Identification Results.

Transfer Function	Thermal Time Constant	NRMSE ^1^
$H_{32}\left(s\right)=\frac{3.75}{s+0.0876}$	11.5	88.79%
$H_{41}\left(s\right)=\frac{0.095}{s+0.0136}$	73.4	66.51%

^1^ Normalized Root Mean Square Error [42].

**Table 2:** Relative Error of the CS method with linear and quadratic fit with respect to SS method.

Symbol	Parameter	Mean Relative Error ${\bar{e_{r}}}_{\%}$		Standard Deviation ${\sigma_{r}}_{\%}$	
		Linear	Quadratic	Linear	Quadratic
$\alpha_{s}$	Seebeck coefficient	4.68	2.63	0.17	0.15
$R_{IN}$	Electrical resistance	3.80	1.65	0.11	0.11
$\Theta_{IN}$	Thermal resistance	5.50	3.03	0.21	0.19
$Z\bar{T}$	Figure of merit	7.64	3.91	0.25	0.24

**Table 3:** TES1-12730 Parameters.

Symbol	Description	Value	
n	Number of thermocouples	127	
A	Single module area [mm] × [mm]	30 × 30	
$T_{h}$	Hot side temperature at environment [°C]	27	50
$\Delta T_{\max}$	Temperature Difference when cooling capacity is zero at cold side [°C]	68	76
$V_{\max}$	Voltage applied to the module at $\Delta T_{\max}$ [V]	15.5	17.4
$I_{\max}$	DC current through the modules at $\Delta T_{\max}$ [A]	3.5	3.5
$Q_{C_{\max}}$	Cooling capacity at cold side of the module under ΔT=0 °C [W]	34.1	37.4
$R_{in}$	Module resistance under AC [Ω]	3.5~3.9	3.8~4.3
$R_{ds}$	Internal resistance ^1^ [Ω]	3.42	3.79
$\alpha_{ds}$	Seebeck coefficient ^1^ [mV/K]	51.7	53.7
$\Theta_{ds}$	Thermal resistance ^1^ [K/W]	3.24	3.27

^1^ Data derived using method in [30].

**Table 4:** Interpolating Functions of the TEM Parameters.

Units	Function	$R^{2}$
$\left[\frac{mV}{K}\right]$	$\alpha_{S}=\frac{\Delta T}{38.55}+\frac{\bar{T_{c}}}{15.52}+48.63$	0.9967
[Ω]	$R_{IN}=\frac{\Delta T}{102.3}+\frac{\bar{T_{c}}}{52.48}+3.256$	1.0000
$\left[\frac{K}{W}\right]$	$\Theta_{IN}=\frac{\Delta T}{57.43}+\frac{\bar{T_{c}}}{10^{8}}+2.929$	0.9992
[−]	$Z\bar{T}=\frac{\Delta T}{255.4}+\frac{\bar{T_{c}}}{673.4}+\frac{\Delta T^{2}\;\bar{T_{c}}}{3.4e6}-\frac{\Delta T\;{\bar{T_{c}}}^{2}}{1.24e6}+0.556$	0.9996
